# Respiratory effects of different recruitment maneuvers in acute respiratory distress syndrome

**DOI:** 10.1186/cc6869

**Published:** 2008-04-16

**Authors:** Jean-Michel Constantin, Samir Jaber, Emmanuel Futier, Sophie Cayot-Constantin, Myriam Verny-Pic, Boris Jung, Anne Bailly, Renaud Guerin, Jean-Etienne Bazin

**Affiliations:** 1 General Intensive Care Unit, Hotel-Dieu Hospital, University Hospital of Clermont-Ferrand, Boulevard L. Malfreyt, 63058 Clermond-Ferrand, France; 2 SAR B, Saint-Eloi Hospital, University Hospital of Montpellier, Avenue Augustin Fliche, 34000 Montpellier, France; 3 Department of Medical Imaging, Hotel-Dieu Hospital, University Hospital of Clermont-Ferrand, Boulevard L. Malfreyt, 63058 Clermond-Ferrand, France

## Abstract

**Introduction:**

Alveolar derecruitment may occur during low tidal volume ventilation and may be prevented by recruitment maneuvers (RMs). The aim of this study was to compare two RMs in acute respiratory distress syndrome (ARDS) patients.

**Methods:**

Nineteen patients with ARDS and protective ventilation were included in a randomized crossover study. Both RMs were applied in each patient, beginning with either continuous positive airway pressure (CPAP) with 40 cm H_2_O for 40 seconds or extended sigh (eSigh) consisting of a positive end-expiratory pressure maintained at 10 cm H_2_O above the lower inflection point of the pressure-volume curve for 15 minutes. Recruited volume, arterial partial pressure of oxygen/fraction of inspired oxygen (PaO_2_/FiO_2_), and hemodynamic parameters were recorded before (baseline) and 5 and 60 minutes after RM. All patients had a lung computed tomography (CT) scan before study inclusion.

**Results:**

Before RM, PaO_2_/FiO_2 _was 151 ± 61 mm Hg. Both RMs increased oxygenation, but the increase in PaO_2_/FiO_2 _was significantly higher with eSigh than CPAP at 5 minutes (73% ± 25% versus 44% ± 28%; *P *< 0.001) and 60 minutes (68% ± 23% versus 35% ± 22%; *P *< 0.001). Only eSigh significantly increased recruited volume at 5 and 60 minutes (21% ± 22% and 21% ± 25%; *P *= 0.0003 and *P *= 0.001, respectively). The only difference between responders and non-responders was CT lung morphology. Eleven patients were considered as recruiters with eSigh (10 with diffuse loss of aeration) and 6 with CPAP (5 with diffuse loss of aeration). During CPAP, 2 patients needed interruption of RM due to a drop in systolic arterial pressure.

**Conclusion:**

Both RMs effectively increase oxygenation, but CPAP failed to increase recruited volume. When the lung is recruited with an eSigh adapted for each patient, alveolar recruitment and oxygenation are superior to those observed with CPAP.

## Introduction

Over the last 15 to 20 years, large gains in our knowledge of acute respiratory distress syndrome (ARDS) and its management have been made [1-4]. It has been clearly established that mechanical ventilation can induce acute lung injury (ALI) by causing hyperinflation of healthy lung regions and repetitive opening and closing of unstable lung units [5]. As a consequence, the therapeutic target of mechanical ventilation in patients with ARDS has shifted from the maintenance of 'normal gas exchange' to the protection of the lung from ventilator-induced lung injury. Reduction of tidal volume (V_T_) to limit plateau pressure (P_plat_) is recommended for the ventilatory management of ARDS [6,7]. However, a reduction in V_T _promotes a decrease in lung aeration [8]. Several studies recommend the adjunction of recruitment maneuvers (RMs) to mechanical ventilation to limit alveolar derecruitment induced by low V_T _[9-11].

Classically, a lung RM requires briefly increasing the alveolar pressure to a level above that recommended during ongoing management of ALI/ARDS, so as to aerate lung units filled with edema or inflammatory cells. According to experimental [4,12,13] and human [14,15] studies, re-aeration of a non-aerated lung unit depends not only on the inflating pressure, but also on the duration of sustained pressure, the so-called inflating pressure-time product (pressure × time) [16]. It follows, then, that for an RM to be effective, its duration should be optimized. We recently reported the efficiency of extended sigh (eSigh) in the management of ARDS [17]. eSighs have been used by other groups [18-20]. To date, there are no data comparing the efficacy and safety of different RMs. The aim of this study was to compare the respiratory effects of two RMs, a continuous positive airway pressure (CPAP) and an eSigh, in patients with ARDS under protective mechanical ventilation. The impact on recruited volume (RV) and gas exchange was specifically addressed.

## Materials and methods

The study was approved by the Institutional Review Board of Clermont-Ferrand, France, and written informed consent was obtained from the patients' next of kin.

### Study population

We studied 19 consecutive unselected patients who met the ARDS criteria of the American European Consensus Conference [21]. Exclusion criteria were refusal of consent, age under 18 years, chronic respiratory insufficiency (chronic obstructive pulmonary disease, asthma, restrictive respiratory insufficiency), intracranial hypertension, bronchopleural fistula, and the persistence of unstable hemodynamics despite appropriate support therapy. Patients were orally intubated, sedated with remifentanil (0.2 to 0.4 μg/kg per minute) and midazolam (4 mg/hour), paralyzed with cis-atracurium (15 mg/hour), and ventilated with an Evita 2 Dura ventilator (Dräger, Lübeck, Germany). All patients were equipped with a radial or femoral arterial catheter (Arrow Inc., Erding, Germany). pH, arterial partial pressure of oxygen (PaO_2_), and arterial partial pressure of carbon dioxide (PaCO_2_) were measured using an IL BGE™ blood gas analyzer (Instrumentation Laboratory, Paris, France). The patients were on volume-controlled mechanical ventilation with a V_T _of 6 mL/kg of dry body weight and the highest respiratory rate allowing the maintenance of a PaCO_2 _of less than or equal to 46 mm Hg without intrinsic positive end-expiratory pressure (PEEP) [10]. The fraction of inspired oxygen (FiO_2_) was set at 1, Ti/Ttot (ratio of time of inspiration to total time of breath) at 33%, and the PEEP at 3 cm H_2_O above the lower inflection point (LIP) of the pressure-volume (P-V) curve [22] or at 10 cm H_2_O in the absence of LIP.

### Study design

Before the beginning of the study, volemic status of the patients was checked according to pulmonary artery catheter (if the patient needed one before study inclusion) or echocardiography. If necessary, fluid administration or vasopressor adaptation was performed. During the protocol, no fluid administration or vasopressor modification was allowed (in the absence of a life-threatening episode).

**Figure 1:**
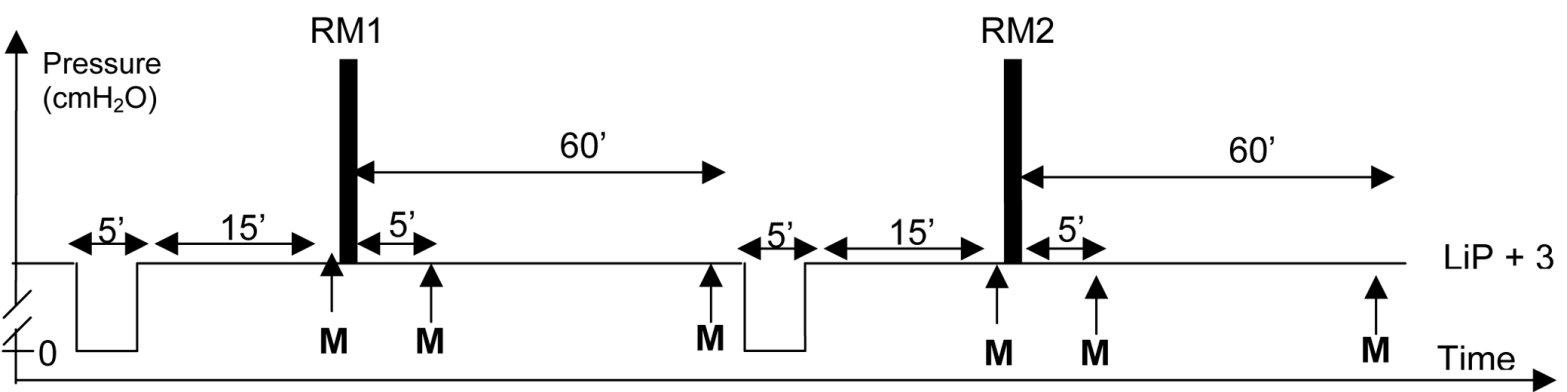
Illustration of the time course of the study. Nineteen patients ventilated with protective lung strategy first had a washout period of 5 minutes of zero end-expiratory pressure ventilation. After 15 minutes of stabilization in positive end-expiratory pressure (PEEP) ventilation, baseline measures (M) were obtained. Then, patients were randomly asssigned to benefit from one of the two recruitment maneuvers (RMs): RM1 or RM2 (that is, continuous positive airway pressure or extended sigh). At 5 and 60 minutes after RM, measurements were obtained. After this first part of the study, a second washout period was performed followed by 15 minutes of ventilation in PEEP and the second RM was performed. The same measurements were performed at baseline and at 5 and 60 minutes after RM. M indicates blood gas analysis, recruited volume by pressure-volume curve method, hemodynamics, and respiratory parameters. LIP, lower inflection point.

Following a 5-minute period of mechanical ventilation in zero end-expiratory pressure (ZEEP), mechanical ventilation was reset with PEEP 3 cm H_2_O above the LIP. Following a 15-minute period of mechanical ventilation in PEEP, cardiorespiratory parameters were recorded and alveolar recruitment was measured by the P-V curve method [17,23-25]. After the collection of these data, patients were randomly assigned to benefit from one of the two RMs. Following the first RM, the patient was ventilated with the initial ventilator settings. Cardiorespiratory and RV measurements were performed 5 and 60 minutes after RM. Before the second RM, a 5-minute period of ZEEP ventilation was performed (return to baseline) followed by a 15-minute period of PEEP ventilation. During both ZEEP periods, if oxygen saturation as measured by pulse oximetry (SpO_2_) decreased below 92%, PEEP ventilation with the PEEP set at the initial value was resumed. After measurements of cardiorespiratory parameters and RV, the second RM was performed (crossover). Five and 60 minutes after this second RM, cardiorespiratory and RV measurements were performed. The time course of the protocol is summarized in Figure 1.

### Recruitment maneuvers

**Figure 2:**
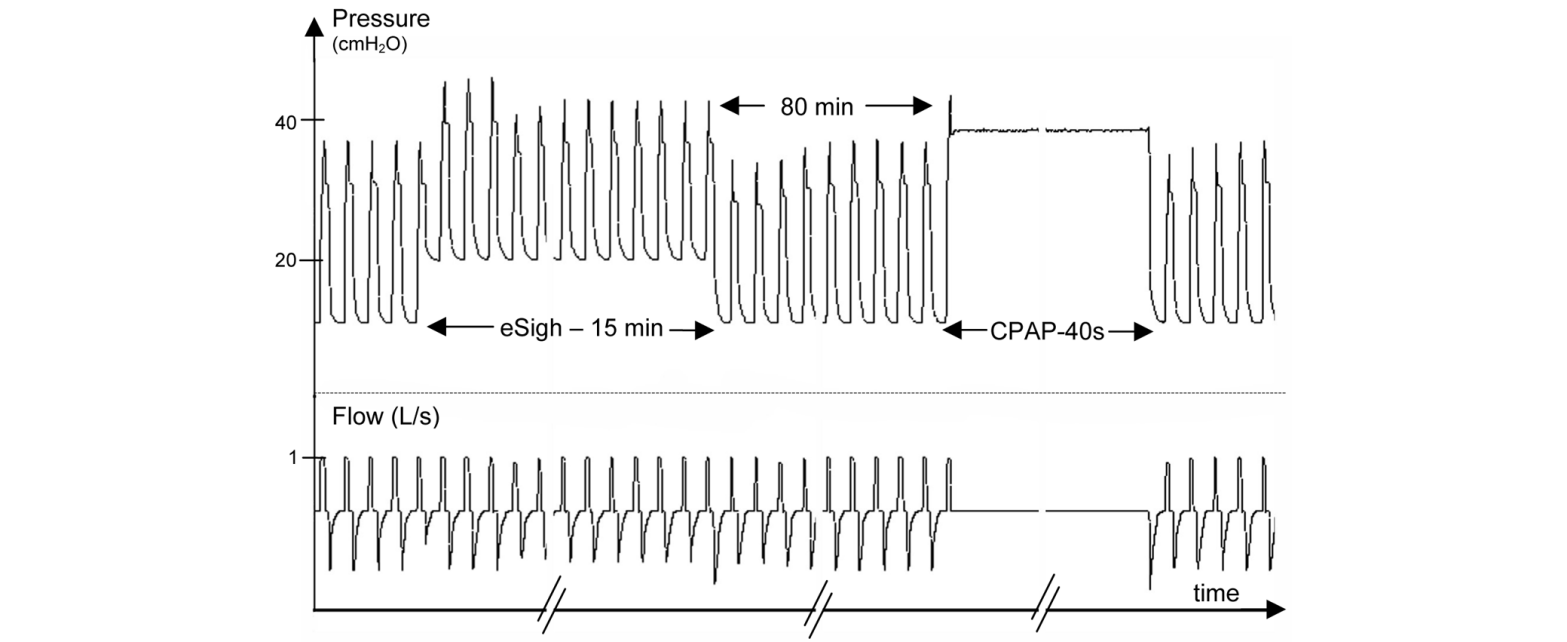
Pressure-time and flow-time curves of a representative patient with a lower inflection point at 11 cm H_2_O and an upper inflection point (UIP) at 39 cm H_2_O. This patient was randomly assigned to benefit from extended sigh (eSigh) first. Initially, positive end-expiratory pressure (PEEP) was set at 14 cm H_2_O and tidal volume (V_T_) at 480 mL. During eSigh, PEEP was increased to 21 cm H_2_O. Plateau pressure was higher than UIP, so V_T _was decreased to 390 mL for 15 minutes. After an 80-minute period (Figure 1), the second recruitment maneuver (RM) (continuous positive airway pressure [CPAP]) was performed at 40 cm H_2_O for 40 seconds. After this second RM, PEEP was set at 14 cm H_2_O. On the flow-time curve, we can see two large expiratory cycles after both RMs corresponding to RM-induced changes in end-expiratory lung volume.

CPAP was performed by imposition of a pressure of 40 cm H_2_O for 40 seconds without V_T _[26,27] (Figure 2a). As previously described [17], our method of performing RM, eSigh, consisted of increasing PEEP 10 cm H_2_O above the LIP for 15 minutes, the patient being on volume-controlled ventilation (Figure 2b). If necessary, V_T _was decreased to maintain P_plat _below the upper inflection point (UIP) or below 35 cm H_2_O if UIP could not be identified on the ZEEP P-V curve. During the RM, the maximum peak airway pressure was limited to 50 cm H_2_O. In case of severe arterial hypotension (systolic arterial pressure of less than 70 mm Hg) or severe hypoxemia (SpO_2 _of less than 80%), the RM was immediately stopped. A positive response to RM was defined *a priori *as a 20% increase in RV 5 or 60 minutes after RM [28].

### Measurement of alveolar recruitment by the pressure-volume curve method

PEEP-induced changes in end-expiratory lung volume (EELV) were measured using a heated pneumotachograph (Hans Rudolph, Inc., Shawnee, KS, USA) positioned between the Y-piece and the connecting piece. The pneumotachograph was previously calibrated by a supersyringe filled with 1,000 mL of air. The precision of the calibration was 3%. The respiratory tubing connecting the endotracheal tube to the Y-piece of the ventilator circuit was occluded by a clamp at end-expiration while the ventilator was disconnected from the patient. The clamp was then released and the exhaled volume measured by the pneumotachograph was recorded on a Macintosh Performa 6400 computer (Apple Computer, Inc., Cupertino, CA, USA) using AcqKnowledge 3.7 software (BIOPAC Systems, Inc., Goleta, CA, USA).

P-V curves of the respiratory system were measured on an Evita 2 Dura ventilator (Dräger) using the low constant flow method as described by Lu and colleagues [22]. During the maneuver, the peak airway pressure was limited to 50 cm H_2_O. P-V curves were measured in ZEEP and PEEP conditions. For each patient, alveolar recruitment was measured using the P-V curve method as follows: the P-V curves in ZEEP and PEEP conditions were constructed. Changes in EELV were then added on each volume that served for constructing the P-V curve in PEEP. The two curves were then placed on the same pressure and volume axes. RV was defined as the difference in lung volume between PEEP and ZEEP at an airway pressure of 15 cm H_2_O [29]. When patients have a diffuse loss of aeration in computed tomography (CT) scan, RV was the EELV following PEEP release [23].

### Thoracic computed tomography scan procedure

Lung scanning was performed in the supine position from the apex to the diaphragm by means of a spiral Tomoscan SR 7000 (Philips, Eindhoven, The Netherlands). All images were observed and photographed at a window width of 1,600 Hounsfield units (HU) and a window level of -600 HU. The exposures were taken at 120 kV and 85 mA without contrast material [30]. By institutional protocol and as previously described, lung scanning was performed at ZEEP by briefly disconnecting the patient from the ventilator (10 to 20 seconds). Electrocardiogram, pulse oxymetry, and systemic arterial pressure were continuously assessed throughout the CT procedure. The lowest value of hemoglobin oxygen saturation allowed during the imaging exam was 85% [31,32].

Qualitative assessment of lung morphology was performed by two independent radiologists (AB and J-MG) by applying the 'CT scan ARDS study group' criteria, which establish three patterns of loss of aeration distribution: focal or lobar, diffuse, and patchy [31]. Loss of aeration was defined as a homogeneous increase of pulmonary parenchyma attenuation obscuring the margins of vessels and airway walls [31]. Patients showing a lobar or segmental distribution of loss of aeration, with the possibility of recognizing the anatomical structures such as the major fissura or the interlobular septa, were classified as having a focal ARDS [31].

### Cardiorespiratory measurements

In each patient, heart rate, systemic arterial pressure, and airway pressure were continuously recorded on the BIOPAC system (BIOPAC Systems, Inc.). Fluid-filled transducers were positioned at the midaxillary line and connected to the arterial catheter. Arterial blood pressures were measured at end-expiration and averaged over five cardiac cycles. The compliance of the respiratory system was calculated by dividing the V_T _by the P_plat _minus intrinsic PEEP.

### Statistical analysis

The statistical analysis was performed using Statview 5.0 software (SAS Institute Inc., Cary, NC, USA). All data are expressed as mean ± standard deviation (SD). Baseline clinical characteristics were compared between RMs using the Student *t *test for parametric data and the Mann-Whitney *U *test for non-parametric data. After the verification of the normal distribution of quantitative data using the Kolmogorov-Smirnov test, changes in cardiorespiratory parameters were analyzed by a two-way analysis of variance for repeated measures (at baseline and 5 minutes and 1 hour after RM) and one grouping factor (RM method: CPAP and eSigh) followed by a Student-Newman-Keuls *post hoc *comparison test. The statistical significance level was fixed at 0.05.

## Results

**Figure 3:**
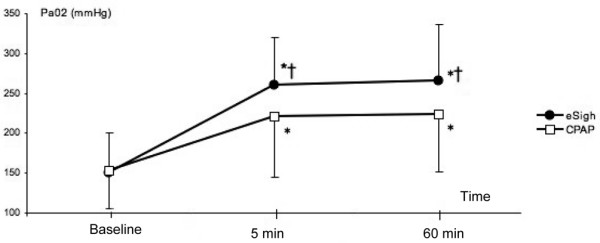
Both recruitment maneuvers increased oxygenation. Extended sigh (eSigh) induced a significantly higher increase in arterial partial pressure of oxygen (PaO_2_) than continuous positive airway pressure (CPAP) at 5 and 60 minutes after the recruitment maneuver. * significant versus baseline, † significant versus CPAP.

**Figure 4:**
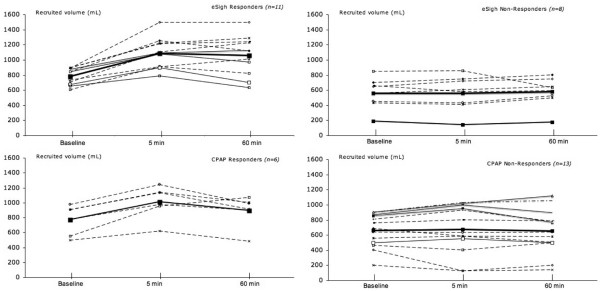
Recruited volume in responders and non-responders according to recruitment maneuver method. Eight patients were non-responders for extended sigh (eSigh) and 13 for continuous positive airway pressure (CPAP). Changes in recruited volume were significantly higher at 5 and 60 minutes with eSigh only.

Two women and 17 men, with an average age of 59 ± 15 years, were included in the study. The reasons for admission to the intensive care unit and the clinical characteristics of the patients are shown in Table 1. The patients had a PaO_2_/FiO_2 _of 151 ± 61 mm Hg and a mean compliance of 28 ± 3 mL/cm H_2_O. All patients had an early ARDS at inclusion with a mean delay between diagnosis to study inclusion of 27 ± 17 hours. Six patients had a focal, 2 a patchy, and 11 a diffuse loss of aeration on CT scan. V_T _was 445 ± 70 mL throughout the study. During eSigh, V_T _was decreased to 390 ± 101 mL, P_plat _increased from 31 ± 4 to 37 ± 2 cm H_2_O, and peak inspiratory pressure (P_max_) increased from 39 ± 6 to 47 ± 6 cm H_2_O. The mean PEEP value was 14 ± 2 cm H_2_O at baseline and 21 ± 2 cm H_2_O during eSigh. Respiratory and hemodynamic parameters before and after RM are shown in Table 2. As shown in Figure 3, both RMs increased oxygenation at 5 minutes (73% ± 36% for eSigh and 44% ± 64% for CPAP; *P *< 0.0001) and at 60 minutes (76% ± 32% versus 31% ± 50%) but only eSigh significantly increased RV at 5 and 60 minutes (21% ± 22%, *P *= 0.0003, and 21% ± 25%, *P *= 0.001, respectively). CPAP increased RV after 5 minutes (8% ± 22%; *P *= 0.01) but not after 60 minutes (2% ± 28%; *P *= 0.17). As shown in Figure 4, 11 patients were considered as recruiters with eSigh (10 with diffuse loss of aeration) and 6 with CPAP (5 with diffuse loss of aeration). During washout periods, SpO_2 _was always maintained above 92%.

**Table 1:** Clinical and respiratory characteristics of the patients at the study entry

RM order^a^	Age, years	Gender	Height, cm	PBW, kg	Cause of ARDS	SAPS II	Delay, hours	V_T_, mL	RR, rpm	LIP, cm H_2_O	UIP, cm H_2_O	Loss of lung aeration^b^	Outcome^c^
A	59	Male	185	90	Sepsis	48	12	480	25	12	35	Focal	D
A	63	Male	175	70	Aspiration	62	12	490	22	13	44	Focal	S
B	78	Male	178	85	Pneumonia	51	24	440	24	12	-	Focal	S
A	74	Male	180	90	Abdominal sepsis	78	24	450	20	13	-	Focal	D
B	38	Male	182	80	Pneumonia	24	12	470	22	9	45	Diffuse	S
B	68	Male	170	72	Pneumonia	80	24	400	24	12	42	Diffuse	D
A	38	Male	188	85	Aspiration	60	12	500	25	12	-	Diffuse	D
B	49	Male	180	80	Pneumonia	33	24	450	21	12	48	Patchy	S
B	28	Male	195	75	Polytrauma	40	24	533	27	12	49	Diffuse	S
A	63	Male	180	82	Aspiration	78	12	450	20	9	46	Diffuse	S
B	57	Male	175	78	Aspiration	22	12	430	20	13	-	Diffuse	S
A	75	Female	163	52	Abdominal sepsis	76	48	340	18	15	40	Diffuse	D
A	76	Male	180	88	Pneumonia	68	48	450	20	7	40	Diffuse	S
B	80	Female	160	48	Pneumonia	58	12	310	26	13	40	Diffuse	D
A	58	Male	185	90	Pneumonia	38	72	480	27	9	39	Patchy	S
B	71	Male	178	80	Abdominal sepsis	55	48	440	21	8	-	Focal	S
B	52	Male	180	80	Sepsis	48	24	450	20	7	36	Diffuse	S
A	54	Male	175	85	Abdominal sepsis	38	36	430	22	15	-	Focal	S
A	43	Male	185	95	Pneumonia	12	24	480	25	9	34	Diffuse	S

^a^Order of application of the two recruitment maneuvers: A for extended Sigh, B for continuous positive airway pressure.

^b^Diffuse, diffuse loss of aeration; Focal, focal loss of aeration; Patchy, patchy loss of aeration.

^c^D, deceased; S, survived.

ARDS, acute respiratory distress syndrome; Aspiration, aspiration pneumonia; Delay, delay between the diagnosis of acute respiratory distress syndrome and inclusion in the study; LIP, lower inflection point on the pressure-volume curve; PBW, predicted body weight; rpm, respirations per minute; RR, respiratory rate; SAPS, simplified acute physiologic score (evaluated at the beginning of the study); UIP, upper inflection point on the pressure-volume curve; V_T_, tidal volume.

**Table 2:** Respiratory and hemodynamic parameters before and after recruitment maneuver

	Extended sigh			Continuous positive airway pressure		
	Baseline	5 minutes	60 minutes	Baseline	5 minutes	60 minutes

Plateau pressure, cm H_2_O	31 ± 4	28 ± 5	28 ± 5	31 ± 3	30 ± 3	30 ± 3
End-expiratory lung volume, mL	834 ± 133	957 ± 228^a^	998 ± 184^a^	927 ± 191	1,097 ± 120^a^	1,001 ± 133^a^
Recruited volume, mL	692 ± 189	867 ± 339^a^	857 ± 335^a^	695 ± 217	781 ± 328^a^	730 ± 288
Quasi-static compliance, mL/cm H_2_O	28 ± 3	36 ± 4^a^	37 ± 4^a^	29 ± 3	32 ± 3	33 ± 3
PaCO_2_, mm Hg	52 ± 12	56 ± 10	55 ± 11	54 ± 9	57 ± 10	55 ± 10
pH	7.28 ± 0.11	7.27 ± 0.08	7.28 ± 0.09	7.28 ± 0.08	7.26 ± 0.09	7.27 ± 0.09
Heart rate, beats per minute	98 ± 22	99 ± 23	99 ± 22	97 ± 22	98 ± 22	98 ± 23
Systolic arterial pressure, mm Hg	123 ± 18	119 ± 10	118 ± 16	125 ± 13	120 ± 16	116 ± 18
Diastolic arterial pressure, mm Hg	62 ± 8	63 ± 9	61 ± 7	64 ± 10	63 ± 8	63 ± 10
Mean arterial pressure, mm Hg	81 ± 12	79 ± 13	80 ± 12	84 ± 10	80 ± 13	81 ± 18

^a^*P *< 0.05 versus baseline. PaCO_2_, arterial partial pressure of carbon dioxide.

**Figure 5:**
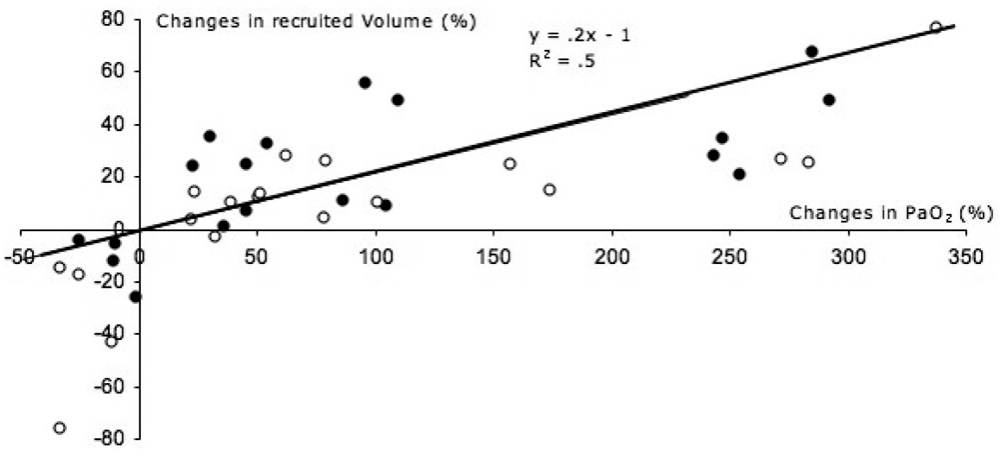
Correlation between recruitment maneuver-induced changes in recruited volume and changes in arterial partial pressure of oxygen (PaO_2_) for extended sigh (full circles) and continuous positive airway pressure (empty circles).

The only significant hemodynamic change was a decrease in mean arterial pressure during CPAP in non-responders from 86 ± 12 to 70 ± 16 mm Hg (*P *= 0.0081); the decrease in blood pressure during eSigh was not significant. During the CPAP maneuver, two patients needed interruption of RM due to a drop in systolic arterial pressure below 70 mm Hg. As shown in Figure 5, a significant correlation was found between RM-induced changes in arterial oxygenation and RM-induced alveolar recruitment, regardless of the method used.

## Discussion

Both RMs increased oxygenation but only eSigh RM increased RV in ARDS patients. Hemodynamically, eSigh RM was better tolerated than CPAP RM and induced a greater and more prolonged increase in arterial oxygenation.

### Methodological considerations

The design of the present study (crossover study with the patient being his own control) required the return to baseline ventilation between each RM (ZEEP for 5 minutes). Such a design raises several questions. Was 5 minutes of ZEEP ventilation long enough to return to control values? Was it safe enough for ARDS patients? Is a short period of ZEEP ventilation really representative of conditions encountered in clinical practice? RV and oxygenation were not different at the two baselines (Table 2 and Figure 4), suggesting that the short period of derecruitment resulting from ZEEP ventilation was long enough to return to comparable conditions before each RM. In each individual patient, the 5-minute period of ZEEP ventilation could be achieved without severe oxygen desaturation imposing the reinstitution of PEEP (as anticipated in the study protocol). In clinical practice, despite the efforts of the medical team to limit episodes of acute derecruitment, such conditions nevertheless occur in patients with ALI: accidental disconnection from the ventilator, open-circuit endotracheal suctioning [33], endobronchial fiberoptic procedure with or without bronchoalveolar lavage, blind mini-bronchoalveolar lavage for the diagnosis of ventilator-associated pneumonia [34], and ventilator malfunction requiring ventilator replacement and changes of tracheostomy tubes and ventilator circuits. We recommend that, following such events, RMs be performed [10,33], and therefore the experimental design of the present study can be considered as of clinical relevance.

In this study, we compared two different RM methods. The first one is the widely used CPAP 40 cm H_2_O for 40 seconds [26,35]. We compared this method with an eSigh performed in volume control ventilation. In previous studies [36,37], a conventional form of sigh was found to be inadequate as a recruitment method in ARDS lungs. Inflating pressure during a conventional sigh, though perhaps sufficient in magnitude, is exerted on the lung only briefly. This brevity of pressure application, in light of current knowledge, would not re-aerate and/or splint lung units with a heightened collapsing tendency [38]. This limitation of a conventional sigh was shown again in a study by Pelosi and colleagues [36], in which the effect of improved oxygenation and decreased lung elastance seen during a sigh period was soon lost after its discontinuation. The PEEP level set after sigh was probably insufficient in this study. Safety and efficacy of an eSigh were established in several studies [11,17,19,39]. As previously reported by our group [17] and in the present study, this method increased alveolar recruitment and oxygenation in ARDS patients without respiratory or hemodynamic complications.

RM-induced changes in hemodynamic parameters were limited to a decrease in arterial pressure during RM in non-responders. But in this study, patients did not benefit from cardiac output monitoring (that is, pulmonary artery catheter or echocardiography). This could underestimate the hemodynamic impact of RM [40]. CPAP interruption, due to a drop in arterial pressure below 70 mm Hg, was required in two patients, whereas eSigh was well tolerated, with a smaller decrease in blood pressure. This adverse event was previously described, but it underscores a major concern for routine use of this procedure. In 16 patients after open heart surgery, Celebi and colleagues [41] have already described this difference between CPAP and high PEEP recruitment methods.

### Recruitment maneuver-induced changes in oxygenation and recruited volume

The present study shows that only eSigh significantly increases RV. Changes in these parameters are more significant than raw data. It must be pointed out that, at baseline, PEEP level was optimized according to the P-V curve. So PEEP-induced alveolar recruitment and EELV were relatively high at baseline; RM-induced RV appears inferior to that obtained with a standardized low PEEP. RV was assessed by the P-V curve method [29]. In a previous study, Lu and colleagues [23] compared this method with the reference method (CT scan) and showed that RV measured by P-V curve is highly correlated with RV measured by CT scan, but the P-V curve method underestimates recruitment in patients with diffuse loss of aeration. When the whole lung is poorly or not aerated, PEEP-induced alveolar recruitment is exactly PEEP-induced changes in EELV. A further study, based on CT measurement of lung recruitment, is required to definitively confirm these results.

As previously demonstrated for PEEP and RM [17,42], a weak but statistically significant correlation was found between RM-induced alveolar recruitment and RM-induced improvement in arterial oxygenation (Figure 5). In fact, alveolar recruitment is an anatomical phenomenon depending exclusively on the penetration of gas into poorly or non-aerated lung regions, whereas arterial oxygenation is a complex physiologic parameter depending on multiple factors such as lung aeration, regional pulmonary flow, mixed venous oxygen saturation, and cardiac index [4].

Changes in RV and increases in oxygenation are higher with eSigh versus CPAP. Different hypotheses may be proposed to explain these facts. First, alveolar recruitment is a time-dependent phenomenon and procedure duration could influence the response to RM. One CPAP may not be sufficient, and perhaps two or three consecutive CPAPs should be used [43]. Second, several studies based on CT scan, P-V curves, or gas exchange have demonstrated that recruitment is a continuous and progressive phenomenon that depends not only on PEEP, but also on peak inflation pressure [44]. eSigh was performed for 15 minutes with 3 cm H_2_O P_plat _below CPAP, but 7 cm H_2_O P_max _above CPAP. A significantly higher P_max _may explain, in part, why 5 patients were CPAP responders whereas 11 were eSigh responders. During mechanical ventilation, a reduction in V_T _decreases lung recruitment [8]. We can hypothesize that RM without V_T _failed to achieve alveolar recruitment. The third point is the pressure level during RM. The use of CPAP as an RM has been described previously [26] using 40 cm H_2_O for all patients. Effective pressure, during RM, is different if PEEP is set at 8 or 18 cm H_2_O. We believe that it is important to have knowledge of the pulmonary mechanics of patients in order to adapt the pressure level for optimal lung recruitment.

In ARDS patients ventilated with a lung-protective strategy, the effects of RM are discussed. In 17 patients with high PEEP and low V_T_, Villagrá and colleagues [39] concluded that RMs have no short-term benefit on oxygenation and that regional alveolar overdistension capable of redistributing blood flow toward non-aerated lung regions can occur during RM. In 22 patients, Grasso and colleagues [45] found an increase in oxygenation and RV with diminished elastance in responders (early ARDS) after RM in patients with lung-protective strategy. PaO_2_/FiO_2 _decreased from 480 mm Hg (2 minutes after RM) to 300 mm Hg 20 minutes later. The mean PEEP value was 9 ± 2 cm H_2_O. In the present study, in which the mean PEEP value was 14 ± 2 cm H_2_O, we found significant effects of RMs and these effects persisted after 1 hour. As previously reported [46], our data suggest that lung morphology predicts the response to RM, but not baseline ventilator strategy or ARDS history [25]. Indeed, patients with a diffuse loss of aeration are responders to RM, whereas non-responders have a focal loss of aeration predominant in the inferior and posterior lung areas [42,47]. In these patients, performing RM could induce overinflation of the previously healthy lung [17]. Moreover, a high level of PEEP is fundamental to ensure the prolonged effect of RM. The mean PEEP was 5 cm H_2_O higher than that of the study performed by Grasso and colleagues [45]. Furthermore, FiO_2 _was set at 1 throughout this study to 'standardize' measurements. In 'real life', a reduction in FiO_2 _will limit oxygen-induced loss of aeration.

## Conclusion

When the lung is recruited with eSigh adapted for each patient, alveolar recruitment and oxygenation are superior to those observed with one CPAP and the hemodynamic tolerance is greater. This study points out the need to adapt the pressure level required for effective RMs. Lung morphology by CT scan and P-V curve should guide the clinician to predict the response to RM and to choose the effective pressure level. The PEEP level post-RM is crucial for maintaining the effect.

## Key messages

• Pulmonary mechanics-based recruitment maneuvers (RMs) (extended sigh, or eSigh) are more efficient than one continuous positive airway pressure.

• Both RMs increased oxygenation but only eSigh increased recruited volume.

• The pressure level required for RM, as positive end-expiratory pressure level after RM, must be adapted for each patient.

## Abbreviations

ALI = acute lung injury; ARDS = acute respiratory distress syndrome; CPAP = continuous positive airway pressure; CT = computed tomography; EELV = end-expiratory lung volume; eSigh = extended sigh; FiO_2 _= fraction of inspired oxygen; HU = Hounsfield units; LIP = lower inflection point; PaCO_2 _= arterial partial pressure of carbon dioxide; PaO_2 _= arterial partial pressure of oxygen; PEEP = positive end-expiratory pressure; P_max _= peak inspiratory pressure; P_plat _= plateau pressure; P-V = pressure-volume; RM = recruitment maneuver; RV = recruited volume; SpO_2 _= oxygen saturation as measured by pulse oximetry; UIP = upper inflection point; V_T _= tidal volume; ZEEP = zero end-expiratory pressure.

## Competing interests

The authors declare that they have no competing interests.

## Authors' contributions

J-MC participated in the design of the study, carried out the study, and drafted the manuscript. SJ participated in the design of the study and helped to draft the manuscript. EF and SC-C participated in the study and study analysis. MV-P participated in the acquisition of study data and helped to draft the manuscript. AB participated in the CT scan analysis and helped in the redaction of the manuscript. RG, BJ, and J-EB participated in the design of the study and helped to draft the manuscript. All authors read and approved the final manuscript.

## References

[B1] Bernard GR (2005). Acute respiratory distress syndrome: a historical perspective. Am J Respir Crit Care Med.

[B2] Terragni PP, Rosboch G, Tealdi A, Corno E, Menaldo E, Davini O, Gandini G, Herrmann P, Mascia L, Quintel M, Slutsky AS, Gattinoni L, Ranieri VM (2007). Tidal hyperinflation during low tidal volume ventilation in acute respiratory distress syndrome. Am J Respir Crit Care Med.

[B3] Rouby JJ (2007). Recruitment in pulmonary and extrapulmonary acute respiratory distress syndrome: the end of a myth?. Anesthesiology.

[B4] Koefoed-Nielsen J, Nielsen ND, Kjaergaard AJ, Larsson A (2008). Alveolar recruitment can be predicted from airway pressure-lung volume loops: an experimental study in a porcine acute lung injury model. Crit Care.

[B5] Dreyfuss D, Saumon G (1998). Ventilator-induced lung injury: lessons from experimental studies. Am J Respir Crit Care Med.

[B6] Wolthuis EK, Veelo DP, Choi G, Determann RM, Korevaar JC, Spronk PE, Kuiper MA, Schultz MJ (2007). Mechanical ventilation with lower tidal volumes does not influence the prescription of opioids or sedatives. Crit Care.

[B7] Villar J, Kacmarek RM, Perez-Mendez L, Aguirre-Jaime A (2006). A high positive end-expiratory pressure, low tidal volume ventilatory strategy improves outcome in persistent acute respiratory distress syndrome: a randomized, controlled trial. Crit Care Med.

[B8] Richard JC, Maggiore SM, Jonson B, Mancebo J, Lemaire F, Brochard L (2001). Influence of tidal volume on alveolar recruitment. Respective role of PEEP and a recruitment maneuver. Am J Respir Crit Care Med.

[B9] Lapinsky SE, Mehta S (2005). Bench-to-bedside review: Recruitment and recruiting maneuvers. Crit Care.

[B10] Rouby JJ, Lu Q (2005). Bench-to-bedside review: Adjuncts to mechanical ventilation in patients with acute lung injury. Crit Care.

[B11] Barbas CS, de Mattos GF, Borges Eda R (2005). Recruitment maneuvers and positive end-expiratory pressure/tidal ventilation titration in acute lung injury/acute respiratory distress syndrome: translating experimental results to clinical practice. Crit Care.

[B12] Gaver DP, Samsel RW, Solway J (1990). Effects of surface tension and viscosity on airway reopening. J Appl Physiol.

[B13] Neumann P, Berglund JE, Mondejar EF, Magnusson A, Hedenstierna G (1998). Effect of different pressure levels on the dynamics of lung collapse and recruitment in oleic-acid-induced lung injury. Am J Respir Crit Care Med.

[B14] Sydow M, Burchardi H, Ephraim F, Zielmann S, Crozier TA (1994). Long term effects of two different ventilatory modes on oxygenation in acute lung injury. Comparison of airway pressure release ventilation and volume controlled inverse ratio ventilation. Am J Respir Crit Care Med.

[B15] Brower RG, Morris A, MacIntyre N, Matthay MA, Hayden D, Thompson T, Clemmer T, Lanken PN, Schoenfeld D (2003). Effects of recruitment maneuvers in patients with acute lung injury and acute respiratory distress syndrome ventilated with high positive end-expiratory pressure. Crit Care Med.

[B16] Marini JJ (1998). A lung-protective approach to ventilating ARDS. Respir Care Clin N Am.

[B17] Constantin JM, Cayot-Constantin S, Roszyk L, Futier E, Sapin V, Dastugue B, Bazin JE, Rouby JJ (2007). Response to recruitment maneuver influences net alveolar fluid clearance in acute respiratory distress syndrome. Anesthesiology.

[B18] Borges JB, Okamoto VN, Matos GF, Caramez MP, Arantes PR, Barros F, Souza CE, Victorino JA, Kacmarek RM, Barbas CS, Carvalho CR, Amato MB (2006). Reversibility of lung collapse and hypoxemia in early acute respiratory distress syndrome. Am J Respir Crit Care Med.

[B19] Lim CM, Koh Y, Park W, Chin JY, Shim TS, Lee SD, Kim WS, Kim DS, Kim WD (2001). Mechanistic scheme and effect of 'extended sigh' as a recruitment maneuver in patients with acute respiratory distress syndrome: a preliminary study. Crit Care Med.

[B20] Lim CM, Jung H, Koh Y, Lee JS, Shim TS, Lee SD, Kim WS, Kim DS, Kim WD (2003). Effect of alveolar recruitment maneuver in early acute respiratory distress syndrome according to antiderecruitment strategy, etiological category of diffuse lung injury, and body position of the patient. Crit Care Med.

[B21] Bernard GR, Artigas A, Brigham KL, Carlet J, Falke K, Hudson L, Lamy M, Legall JR, Morris A, Spragg R (1994). The American-European Consensus Conference on ARDS. Definitions, mechanisms, relevant outcomes, and clinical trial coordination. Am J Respir Crit Care Med.

[B22] Lu Q, Vieira S, Richecoeur J, Puybasset L, Kalfon P, Coriat P, Rouby JJ (1999). A simple automated method for measuring pressure-volume curve during mechanical ventilation. Am J Resp Crit Care Med.

[B23] Lu Q, Constantin JM, Nieszkowska A, Elman M, Vieira S, Rouby JJ (2006). Measurement of alveolar derecruitment in patients with acute lung injury: computerized tomography versus pressure-volume curve. Crit Care.

[B24] Koutsoukou A, Bekos B, Sotiropoulou C, Koulouris NG, Roussos C, Milic-Emili J (2002). Effects of positive end-expiratory pressure on gas exchange and expiratory flow limitation in adult respiratory distress syndrome. Crit Care Med.

[B25] Thille AW, Richard JC, Maggiore SM, Ranieri VM, Brochard L (2007). Alveolar recruitment in pulmonary and extrapulmonary acute respiratory distress syndrome: comparison using pressure-volume curve or static compliance. Anesthesiology.

[B26] Lachmann B (1992). Open up the lung and keep the lung open. Intensive Care Med.

[B27] Amato MB, Barbas CS, Medeiros DM, Magaldi RB, Schettino GP, Lorenzi-Filho G, Kairalla RA, Deheinzelin D, Munoz C, Oliveira R, Takagaki TY, Carvalho CR (1998). Effect of a protective-ventilation strategy on mortality in the acute respiratory distress syndrome. N Engl J Med.

[B28] Constantin JM, Cayot-Constantin S, Roszyk L, Futier E, Sapin V, Bazin JE, Rouby JJ (2007). The response to recruitment maneuver influences net alveolar fluid clearance in acute respiratory distress syndrome. Anesthesiology.

[B29] Ranieri VM, Eissa NT, Corbeil C, Chassé M, Braidy J, Matar N, Milic-Emili J (1991). Effects of positive end-expiratory pressure on alveolar recruitment and gas exchange in patients with the Adult respiratory distress syndrome. Am Rev Resp Dis.

[B30] Bouhemad B, Richecoeur J, Lu Q, Malbouisson LM, Cluzel P, Rouby JJ (2003). Effects of contrast material on computed tomographic measurements of lung volumes in patients with acute lung injury. Crit Care.

[B31] Puybasset L, Cluzel P, Gusman P, Grenier P, Preteux F, Rouby JJ (2000). Regional distribution of gas and tissue in acute respiratory distress syndrome. I. Consequences for lung morphology. CT Scan ARDS Study Group. Intensive Care Med.

[B32] Malbouisson LM, Puybasset L, Constantin JM, Lu Q, Cluzel P, Rouby JJ (2001). Comparison of two CT Scan methods to asses alveolar recruitment in ards. Am J Respir Crit Care Med.

[B33] Lasocki S, Lu Q, Sartorius A, Fouillat D, Remerand F, Rouby JJ (2006). Open and closed-circuit endotracheal suctioning in acute lung injury: efficiency and effects on gas exchange. Anesthesiology.

[B34] Rouby JJ, Rossignon MD, Nicolas MH, Martin de Lassale E, Cristin S, Grosset J, Viars P (1989). A prospective study of protected bronchoalveolar lavage in the diagnosis of nosocomial pneumonia. Anesthesiology.

[B35] Amato MB, Barbas CS, Medeiros DM, Schettino Gde P, Lorenzi Filho G, Kairalla RA, Deheinzelin D, Morais C, Fernandes Ede O, Takagaki TY (1995). Beneficial effects of the 'open lung approach' with low distending pressures in acute respiratory distress syndrome. A prospective randomized study on mechanical ventilation. Am J Respir Crit Care Med.

[B36] Pelosi P, Cadringher P, Bottino N, Panigada M, Carrieri F, Riva E, Lissoni A, Gattinoni L (1999). Sigh in acute respiratory distress syndrome. Am J Respir Crit Care Med.

[B37] Novak RA, Shumaker L, Snyder JV, Pinsky MR (1987). Do periodic hyperinflations improve gas exchange in patients with hypoxemic respiratory failure?. Crit Care Med.

[B38] Marini JJ, Amato MB, Marini JJ, Evans TW (1998). Lung recruitment during ARDS. Acute Lung Injury.

[B39] Villagrá A, Ochagavía A, Vatua S, Murias G, Del Mar Fernández M, Lopez Aguilar J, Fernández R, Blanch L (2002). Recruitment maneuvers during lung protective ventilation in acute respiratory distress syndrome. Am J Respir Crit Care Med.

[B40] Toth I, Leiner T, Mikor A, Szakmany T, Bogar L, Molnar Z (2007). Hemodynamic and respiratory changes during lung recruitment and descending optimal positive end-expiratory pressure titration in patients with acute respiratory distress syndrome. Crit Care Med.

[B41] Celebi S, Koner O, Menda F, Korkut K, Suzer K, Cakar N (2007). The pulmonary and hemodynamic effects of two different recruitment maneuvers after cardiac surgery. Anesth Analg.

[B42] Malbouisson LM, Muller JC, Constantin JM, Lu Q, Puybasset L, Rouby JJ (2001). Computed tomography assessment of positive end-expiratory pressure-induced alveolar recruitment in patients with acute respiratory distress syndrome. Am J Respir Crit Care Med.

[B43] Fujino Y, Goddon S, Dolhnikoff M, Hess D, Amato MB, Kacmarek RM (2001). Repetitive high-pressure recruitment maneuvers required to maximally recruit lung in a sheep model of acute respiratory distress syndrome. Crit Care Med.

[B44] Hickling KG (1998). The pressure-volume curve is greatly modified by recruitment. A mathematical model of ARDS lungs. Am J Respir Crit Care Med.

[B45] Grasso S, Mascia L, Del Turco M, Malacarne P, Giunta F, Brochard L, Slutsky AS, Marco Ranieri V (2002). Effects of recruiting maneuvers in patients with acute respiratory distress syndrome ventilated with protective ventilatory strategy. Anesthesiology.

[B46] Gattinoni L, Caironi P, Cressoni M, Chiumello D, Ranieri VM, Quintel M, Russo S, Patroniti N, Cornejo R, Bugedo G (2006). Lung recruitment in patients with the acute respiratory distress syndrome. N Engl J Med.

[B47] Puybasset L, Gusman P, Muller JC, Cluzel P, Coriat P, Rouby JJ (2000). Regional distribution of gas and tissue in acute respiratory distress syndrome. III. Consequences for the effects of positive end-expiratory pressure. CT Scan ARDS Study Group. Adult Respiratory Distress Syndrome. Intensive Care Med.

